# A mutant retroviral receptor restricts virus superinfection interference and productive infection

**DOI:** 10.1186/1742-4690-9-51

**Published:** 2012-06-12

**Authors:** Meihong Liu, Maribeth V Eiden

**Affiliations:** 1 Section on Directed Gene Transfer, Laboratory of Cellular and Molecular Regulation, National Institute of Mental Health, National Institutes of Health, Bethesda, MD, 20892, USA; 2 Section on Molecular Virology, Laboratory of Cellular and Molecular Regulation, National Institute of Mental Health, National Institutes of Health, Bethesda, MD, 20892, USA

## Abstract

**Background:**

Both cell-free and cell-associated infection routes are important for retroviral dissemination. Regardless of the mechanism, the driving force of retroviral entry is the interaction between the viral envelope and its receptor. To date it remains unclear how decreased affinity of viruses for their receptors affects viral cell-free infection, cell-cell transmission, and spreading kinetics. We have previously characterized a mutant form of the amphotropic murine retrovirus receptor human phosphate transporter 2 (PiT2) wherein the single substitution of a glutamic acid for the lysine residue at position 522 of this receptor is sufficient to render it to function as a gibbon ape leukemia virus (GALV) receptor.

**Results:**

In this study we analyzed the binding affinity of the mutant receptor PiT2K522E and determined that it has a 1000 fold decreased GALV envelope binding affinity compared to the GALV wild type receptor. The decreased affinity does not restrict the initiation of cell-free GALV infection. The diminished binding affinity does, however, correlate with a decrease in the ability of GALV to spread in cells expressing this mutant receptor.

**Conclusions:**

The reduced ability of GALV to subsequently spread among cells expressing PiT2K522E is likely resulted from reduced cell-cell transmission, the decreased ability of PiT2K522E-expressing cells to establish superinfection interference, and attendant cytopathic affects.

## Background

Both cell-free and cell-associated infections are important for retroviral dissemination. However, cell-associated viral infection is over a thousand fold more efficient in vivo [1]. To enter cells, cell-free enveloped viruses bind to specific receptors on the target cell surface. They then penetrate the host cells by either direct fusion of viral and cellular lipid membranes or via an endocytotic pathway. Both entry pathways result in the release of the viral nucleocapsid into the cytoplasm [2-4]. Several mechanisms have been invoked for the transmission of virus from an infected cell to an uninfected cell. All involve the interaction of viral envelope in the membrane of the infected cell with the receptor on the uninfected target cell triggering a signal that causes cytoskeleton rearrangement. Several adhesion molecules are recruited to participate at the cell contact site to form a virological synapse and filopodial bridges [5-7].

The affinity thresholds that accompany the association of viral envelope proteins in the membrane of infected cells with their receptors required to trigger viral entry vary greatly. Influenza virus requires millimolar range affinity while human immunodeficiency virus (HIV) requires binding affinity in the nanomolar range [1]. Higher affinities result in more efficient binding of a single viral particle to recruit several receptors thereby accelerating post-binding events that lead to membrane fusion and enhance efficiencies of viral entry. In cell-associated infection routes, envelope proteins are highly enriched on the infected cell interface. This enrichment allows more efficient recruitment of receptors and subsequent access to signaling proteins at levels that make cells more susceptible to viral replication. For example, this enrichment facilitates the utilization of actin-driven motion [7] and cellular proteins that interact with the host cell cytoskeleton [8] to support intracellular transport and membrane fusion events associated with viral entry. Decreased affinity between viral envelope and receptor has been reported to cause delayed viral replication kinetics and is linked to the failure to establish superinfection resistance, apoptosis, and induce syncytia formation [9-12]. However, it remains unclear how a decreased receptor affinity affects viral cell-associated infection and spreading kinetics.

As gammaretroviruses, amphotropic murine leukemia virus (A-MLV) and gibbon ape leukemia virus (GALV) have divergent host ranges and are not in the same interference class [13]. The receptors for GALV and A-MLV encode distinct but related proteins originally designated GLVR1 and GLVR2 [13]. Later, the GALV and A-MLV receptors were identified to function as type III inorganic phosphate transporters and renamed as PiT1 and PiT2 and are now referred to as SLC20A1 and SLC20A2 in accordance with their transporter classification. Herein, we use the PiT1 and PiT2 nomenclature for ease of cross-referencing. Previously, we reported that the PiT2 ortholog expressed on hamster E36 cells, HaPiT2, in contrast to the human form of the A-MLV receptor (PiT2), functions as a receptor not only for A-MLV, but also GALV [14]. Based on comparison of the deduced amino acid sequences of the HaPiT2 and PiT2 proteins, it was eventually determined that the substitution of a single amino acid residue, glutamate (glutamic acid), for lysine residue at position 522 is sufficient to render PiT2 functional as a GALV receptor while retaining A-MLV receptor function. The titer of GALV enveloped retroviral vector is reduced 5 to 6 fold in cells expressing the mutant receptor PiT2K522E compared to those expressing the GALV receptor PiT1 [15,16].

Although both PiT1 and PiT2K522E function to efficiently mediate transduction by GALV enveloped vectors, the ability of PiT2K522E to function as a receptor for replication-competent GALV has not been previously characterized. Surprisingly, PiT1 and PiT2K522E function very differently in their ability to bind GALV envelope, establish superinfection resistance and facilitate efficient GALV spread following exposure to cell-free virus.

## Results

### Productive infection by GALV is severely restricted in cells expressing PiT2K522E compared to cells expressing PiT1

**Figure 1:**
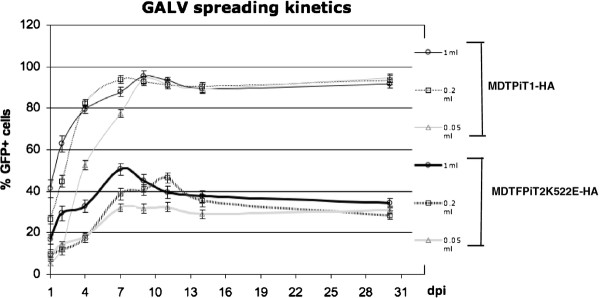
**The kinetics of GALV replication in MDTF cells expressing HA epitope tagged PiT1 or PiT2K522E.** MDTFPiT1-HA (upper three lines) or MDTFPiT2K522E-HA (lower three lines) cells were exposed to 1ml (o), 0.2ml (□) or 0.05ml (▵;) of GALV-GFP viral supernatant in the presence of polybrene. Cells were harvested for flow cytometry analysis at day 1, 2, 4, 7, 9, 11, 14 and 30. The percentage of GFP positive cells is displayed at each time point, day postinfection (dpi). The experiments were repeated three times, and values are presented as average standard deviation.

Although both PiT1 and PiT2K522E confer susceptibility to GALV vectors, the ability of PiT2K522E to support infection by replication-competent GALV has not been evaluated. We, therefore, assessed the capacity of MDTF cells expressing PiT2K522E compared to PiT1 receptors to facilitate viral spread after exposure to cell-free virus. Replication-competent, GFP expressing virus with a GALV envelope [17] was used to assess GALV cell-to-cell transmission *in vitro*. MDTF cells are resistant to GALV. MDTF cells expressing PiT1 or PiT2K522E were exposed to 0.05, 0.2 or 1 ml of supernatant from productively infected GALV-GFP 293 T cells. Every 2 to 3 days, cells were harvested for flow cytometry (FACS) analysis and then further subcultured. As shown in Figure 1, GALV spread rapidly in MDTFPiT1-HA cells. Around 4–5% of MDTFPiT1-HA cells exposed to 0.05 ml GALV-GFP were infected 24 h post-exposure, and maximum infection (93%) was reached at 9 days post-exposure. When the viral inoculums were increased to 0.2 ml or 1 ml, the percent of cells infected correspondingly increased to 26% and 41% at 24 h post-exposure, and maximum infection was reached by day 7 or 9 (Figure 1). GALV spread in cells expressing K522EPiT2 did not exhibit an ability similar to that shown in cells expressing PiT1 where >90% of the cells were productively infected. Infection of MDTF cells expressing the mutant receptor resulted in an initial relatively slow spread of virus in the cultures culminating in a peak of infected cells at day 7 to 9 depending on the initial inoculums (Figure 1). On the first day post exposure, only 3%, 9% or 16% of MDTF cells expressing PiT2K522E were infected when exposed to 0.05, 0.2 or 1 ml GALV-GFP supernatant, respectively. Infection peaked with approximately 40% of cells infected with GALV. After this peak at day 7 or 9, the number of GALV-GFP infected MDTFPiT2K522E-HA cells gradually diminished. To determine if the mutant receptor instability restricts GALV spread, we examined PiT2K522E expression on the surface of MDTF cells by flow cytometry analysis 3 weeks after GALV-GFP exposure. PiT2K522E protein remains stably expressed on the surface of both uninfected and infected cells (Additional file 1: Figure  S1). This result indicates that restriction of GALV spreading is not due to PiT2K522E instability.

Even though CHOK1, like E36 cells, are derived from Chinese hamsters, these two cell types differ in their resistance to infection by GALV: CHOK1 cells are resistant to GALV [14,18]. CHOK1 cells (Chinese hamster kidney cells) expressing either PiT1 or PiT2K522E employed in parallel studies showed similar results (data not shown).

### PiT2K522E exhibits a markedly reduced ability to bind GALV compared to wild type PiT1

**Figure 2:**
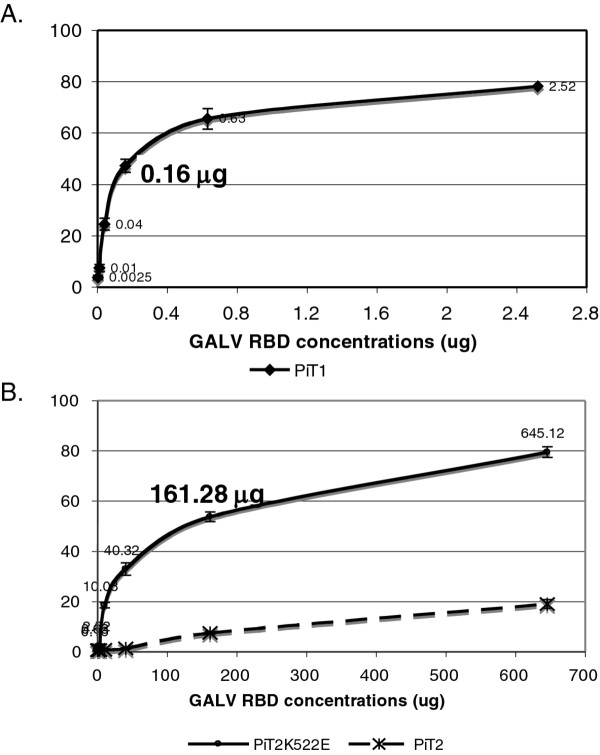
**The binding affinity of GALV RBD with MDTF cells expressing HA tagged PiT1, PiT2K522E or PiT2.** GALV RBD tagged with both V5 and His was purified using high-performance nickel-NTA (Ni-NTA) agarose, serially diluted four times and applied to a binding assay for MDTFPiT1-HA (**A**) or MDTFPiT2K522E-HA and MDTFPiT2-HA cells (**B**). The kinetics of binding of GALV RBD was compared. The mean fluorescence intensity (MFI) for GALV RBD binding is indicated on the *y*-axis and GALVRBD concentration (mg) on the *x*-axis. Three independent experiments were performed and the mean ± standard deviation was presented in the curve.

To further characterize the unusual features of this mutant receptor, we performed SU (the surface component of mature envelope protein) binding assays. Full length GALV SU, in contrast to MLV and FeLV SUs, is not expressed as a functional protein [15]. However, a smaller subunit of the GALV SU encoding the first 219 residues of processed SU and encompassing the receptor binding domain (RBD) and proline rich region (PRR) is expressed and specifically binds PiT1 [15]. We, therefore, employed this 219 residue GALV RBD bearing a V5 epitope tag to detect GALV binding to cells expressing various receptors. Murine MDTF cells are resistant to GALV whereas MDTF cells expressing PiT1 or PiT2K522E are susceptible to GALV. GALV RBD was purified, serially diluted and incubated with MDTF cells expressing hemagglutinin (HA) epitope tagged PiT1, PIT2K522E or PiT2 as described in Materials and Methods. As shown in Figure 2A, 0.16μg of GALV RBD was sufficient to obtain 50% binding to MDTFPiT1-HA cells. In contrast, 160μg of purified GALV RBD was required to achieve similar binding to MDTFPiT2K522E-HA cells (Figure 2B). Thus, a thousand-fold increase in GALV RBD is required to achieve similar binding with cells expressing PiT2K522E-HA compared to MDTFPiT1-HA cells.

Nonspecific binding of murine leukemia virus (MLV) particles to cells has been previously reported [19-21]. To determine if the binding of GALV to MDTF cells expressing PiT2K522E is nonspecific, we examined GALV RBD binding to MDTF cells expressing PiT2 that are not susceptible to GALV infection. As shown in Figure 2B, binding of GALV RBD to MDTF cells expressing PiT2 was significantly lower than that achieved with MDTF cells expressing PiT2K522E. These results indicate that MDTF cells expressing PiT2K522E bind to GALV RBD at a substantially reduced affinity compared to MDTF cells expressing PiT1, but still at a level sufficient to facilitate GALV entry.

### Cells expressing mutant receptor are not deficient in their ability to release infectious particles

**Figure 3:**
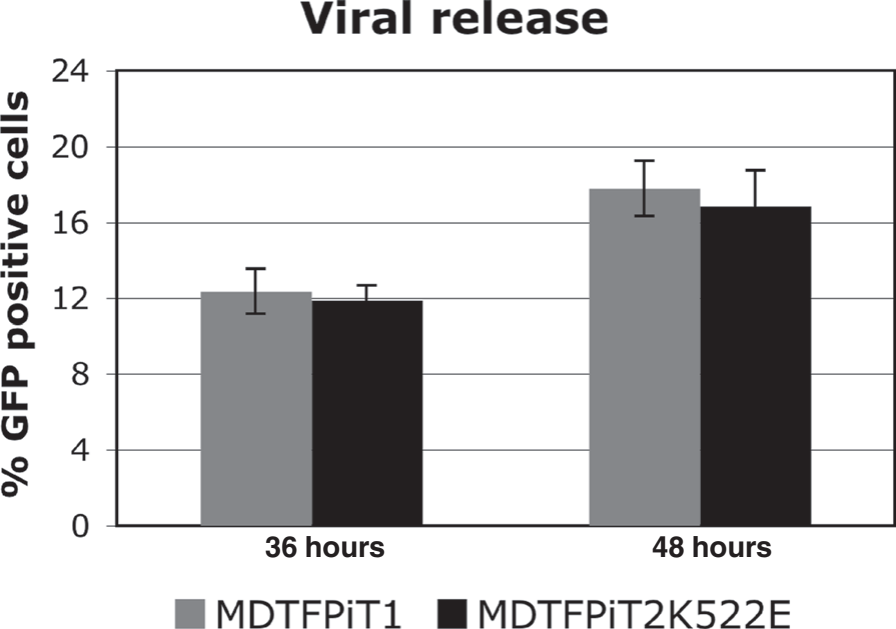
**GALV is release efficiency from MDTF cells expressing PiT1 or PiT2K522E.** MDTF cells expressing receptor PiT1 or PiT2K522E were incubated with 0.5ml GALV-GFP viral stock at 37°C for 1 hour, then the cells were extensively washed and maintained in culture. Media were collected at two time points, 36 and 48 hours after initial exposure to GALV. The titer of GALV-GFP viruses present in 36 hours or 48 hours samples, were assayed by exposing MDTF cells expressing PiT1 to supernatant and performing FACS analysis to determine the percentage of GALV-GFP infected cells at 30 hours after viral exposure. The experiments were repeated three times independently and averaged values are shown.

To investigate whether the decrease in the ability of GALV to spread in cells expressing PiT2K522E is caused by deficiencies in viral particle assembly or release, we performed viral release assays as described in Materials and Methods. MDTF cells expressing PiT1 or PiT2K522E were incubated with GALV-GFP viruses at 37°C for 1 h, and then the cells were extensively washed and maintained in culture. Media were collected at two time points, 36 and 48 h after initial exposure to replication competent GALV-GFP and the titer of the released virus present in supernatant was assessed by exposing MDTF cells expressing PiT1 to supernatant and 30 h later performing FACS analysis to determine the percentage of GALV-GFP infected cells. As shown in Figure 3, the titer of viruses released from GALV-GFP infected MDTF cells expressing PiT1 or PiT2K522E was similar based on the percentage of GFP positive cells. Thus, decreased viral receptor binding affinity does not affect infectious particle assembly or release.

### Decreased GALV binding affinity to the mutant receptor leads to failure to establish superinfection resistance

**Figure 4:**
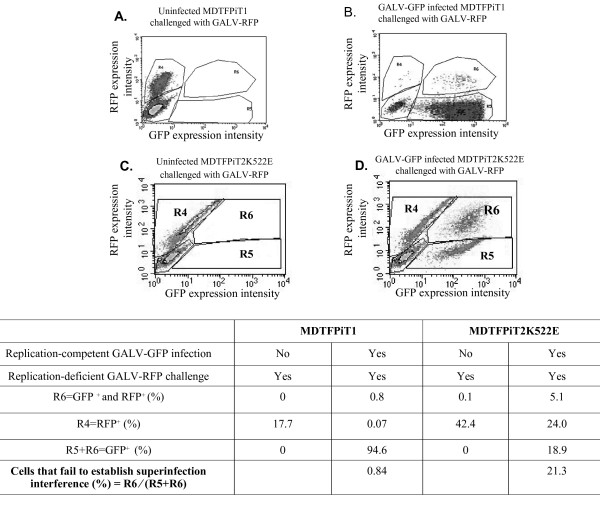
**MDTF cells expressing PiT2K522E cells fail to establish GALV superinfection resistance in.** MDTF cells mock infected or infected with GALV-GFP for two weeks, were challenged with GALV enveloped retroviral vector expressing the fluorescent marker protein, cherry red (GALV-RFP). Two days later, cells were harvested for FACS analysis. MDTFPiT1 cells uninfected (**A**) and infected with GALV-GFP (**B**), MDTFPiT2K522E cells uninfected (**C**) or infected with GALV-GFP (**D**). The *x-*axis represents MFI of GFP expression and the *y*-axis represents MFI of RFP expression. Three experiments were performed and representative images were presented. Region statistics was also exhibited as R3, R4 and R5. R3 = GFP negative and RFP negative, R6 = GFP positive and RFP positive and R5 = GFP positive but RFP negative. The statistics are listed in the table below the figure. The percentage of cells that fail to establish superinfection resistance was calculated by the value in R6 divided by the total values in R6 and R5.

It has been previously reported that a decreased affinity of interaction between viral envelope protein and receptor results in apoptosis, syncytia formation or cytopathic effects (CPE) due to a failure to establish superinfection protection [9]. Therefore, we designed an experiment to assess whether the decreased affinity of mutant receptor results in diminished superinfection resistance. MDTFPiT1 cells and MDTFPiT2K522E cells were infected with GALV-GFP. Two weeks later, they were exposed to GALV enveloped vectors expressing the fluorescent marker protein cherry red (GALV-RFP). As shown in Figure 4A and 4B, only 0.84% GALV-GFP infected MDTFPiT1 were susceptible to GALV cherry red vectors. In comparison, 21.3% of MDTFPiT2K522E cells infected with GALV-GFP remained susceptible to GALV-RFP vectors (Figure 4C and 4D), demonstrating a failure to establish superinfection interference in GALV infected MDTFPiT2K5222K. These results are summarized in the table at the bottom of Figures 4.

### GALV transmission from infected MDTFPiT1K522E to uninfected cells expressing PiT2K522E tagged with HA is not defective but is diminished

**Figure 5:**
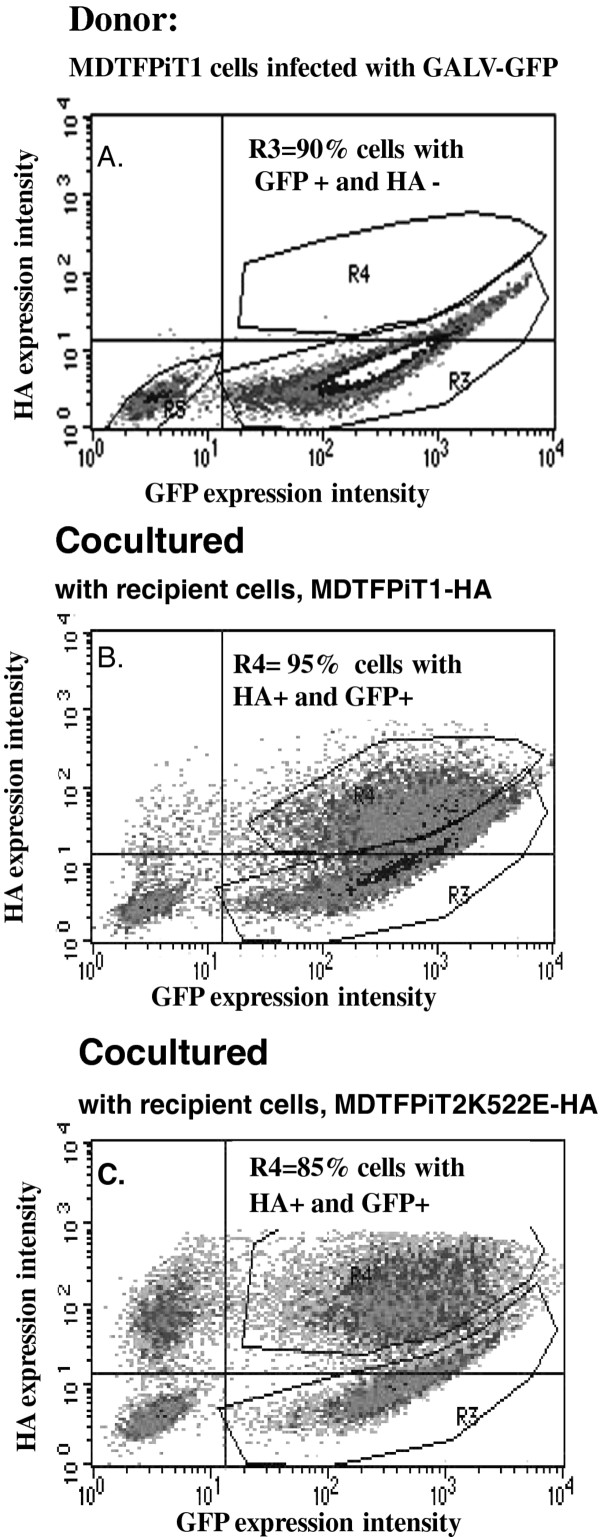
**Evaluation of the efficiency of GALV cell-cell transmission in co-culture experiments with MDTFPiT1 cells productively infected with GALV expressing GFP (GALV-GFP).** MDTFPiT1 cells were exposed to GALV-GFP and then maintained and cultured for one week before being analyzed by FACS for GFP expression (**A**). Next GALV-GFP producer cells were co-cultured with MDTF cells expressing an HA epitope tagged PiT1 and analyzed by FACS for GFP and HA expression (**B**) or MDTF cells expressing the HA epitope tagged mutant receptor PiT2K522E and analyzed by FACS for GFP and HA expression (**C**). Approximately 24 hours after co-culture, cells were harvested for FACS analysis. The MFI of HA expression is indicated on the y-axis and the MFI of GFP expression is on the x-axis. Three independent experiments were performed and representative images presented.

**Figure 6:**
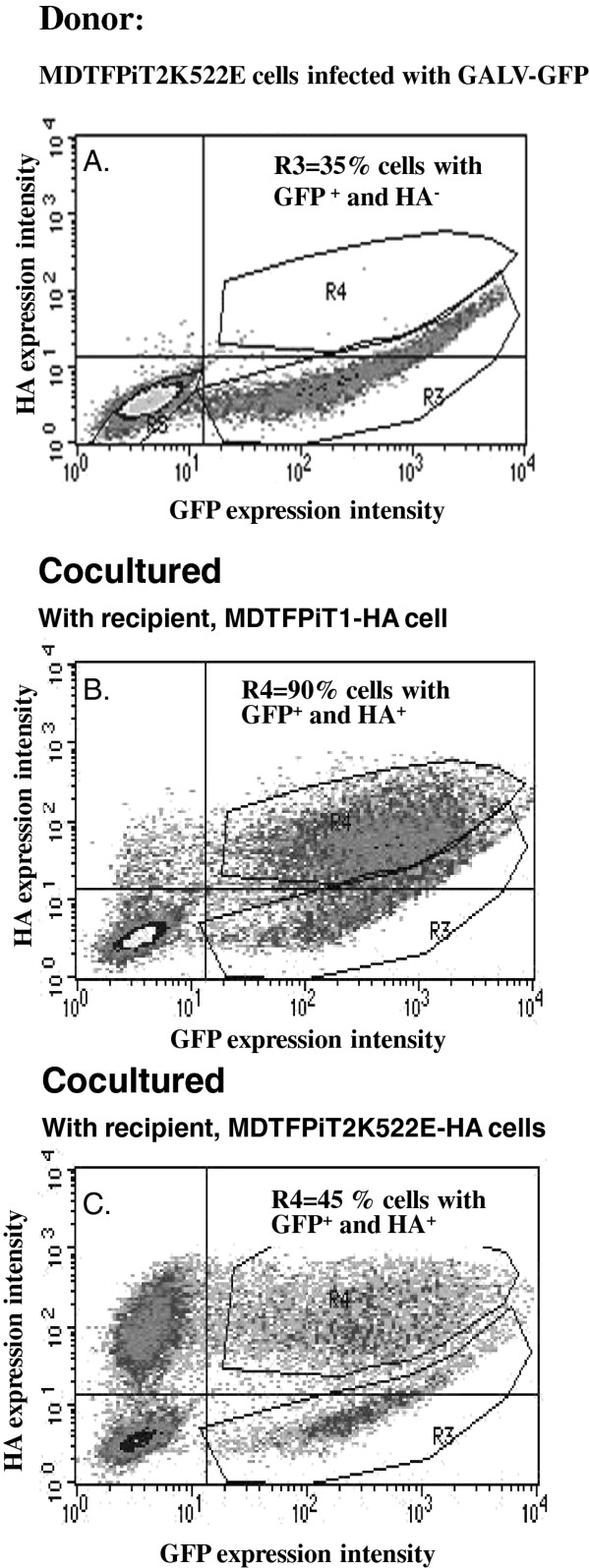
**Efficiency of GALV cell –cell transmission in co-culture experiments where MDTFPiT2K522E cells infected with GALV (A) were used as GALV producing cells, and co-cultured with MDTFPiT1-HA (B) or MDTFPiT2K522E-HA (C).** Three independent experiments were performed and representative images were presented.

To further resolve whether the reduced number of MDTFPiT2K522E cells productively infected with GALV was due to defective cell-cell transmission, we assessed whether GALV can be efficiently delivered to MDTF cells expressing PiT2K522E by co-culturing these cells with cells productively infected with GALV. Co-culture has historically been used to propagate several retroviruses and study viral cell-cell transmission pathways [22]. To evaluate whether GALV infected cells can transmit virus to uninfected MDTFPiT2K522E, we performed a co-culture experiment. MDTF cells expressing PiT1 were exposed to GALV-GFP and grown for one week, and then FACS analysis was performed. About 90% of the target cells were infected (GFP positive, Figure 5A). GALV infected MDTFPiT1 cells were then co-cultured with recipient MDTF cells expressing HA tagged PiT1 or PiT2K522E receptors, at a one-to-one ratio. Twenty-four hours post-exposure to virus infected cells, the cells were harvested, stained with monoclonal HA antibody to detect the HA-tagged viral receptors and analyzed by flow cytometry. As shown in Figure 5 and summarized in Table 1, GALV was transmitted to 95% of MDTFPiT1-HA target cells (Figure 5B) and to 85% of the MDTFPiT2K522E-HA target cells (Figure 5C). The spread of GALV-GFP to both populations of target cells indicates that cells expressing mutant PiT2K522E receptor can be efficiently infected when co-cultured with GALV-producing MDTFPiT1 cells. Next, we investigated whether GALV infected cells expressing mutant PiT2K522E receptors can efficiently transmit GALV to MDTF cells expressing HA-tagged PiT1 or PiT22K522E. As shown in Figure 6, even though only 35% of MDTFPiT2K522E cells were productively infected with GALV (Figure 6A), the virus was efficiently transmitted to MDTFPiT1 target cells in a co-culture experiment (90% recipient cells were infected) (Figure 6B) whereas only 45% of MDTFPiT2K522E target cells were infected following a similar co-culture experiment (6C). These results, summarized in Table 1, suggest that productively infected MDTFPiT2K522E cells can efficiently transmit GALV to target cells expressing PiT1 but are substantially less efficient at transmitting virus to target cells expressing PiT2K552E. Thus cell-cell transmission of GALV infected MDTFPiT2K522E to cells expressing the mutant PiT2K522E is diminished when compared to transmission of GALV from cells expressing PiT1.

**Table 1:** Summary of coculture experiments

GALV production cells	recipient	% recipient infected
MDTFPiT1 infected with GALV-GFP (90% GFP+)	MDTFPiT1-HA	95%
	MDTFPiT2K522E-HA	85%
MDTFPiT2K522E infected with GALV-GFP (35% GFP+)	MDTFPiT1-HA	90%
	MDTFPiT2K522E-HA	45%

### Virological synapses were observed at a reduced frequency in GALV infected cells expressing PiT2K522E compared to those expressing PiT1

**Figure 7:**
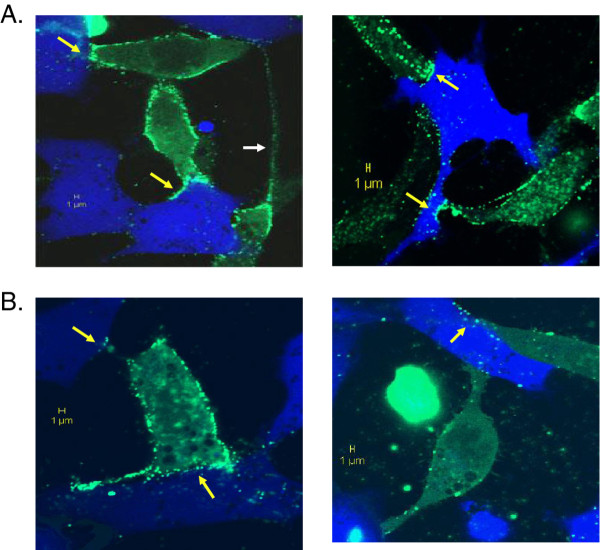
**Gag accumulates at the contact site of GALV producing cells and recipient cells.** GALV infected 293T cells were transfected with the YFP labeled gag (Gag-YFP) expressing construct and, 16 hours post transfection, used as donor cells and co-cultured with recipient, MDTFPiT1-HA cells (**A**) or MDTFPiT2K522E-HA cells (**B**) labeled with CMAC (blue). After being co-cultured for 6 hours, the cells were examined for virological synapse formation (yellow arrows) by confocal microscopy. The Gag-YFP proteins (green) are recruited to the donor and recipient contract site and used for viral assembly so that the GALV particles containing YFP labeled gag (green) are produced and delivered to recipient cells.

The interaction between viral envelope glycoprotein present on infected cells and receptors on uninfected cells drives gag accumulation, triggers de novo viral assembly at the cell-cell contact site and subsequently transfers de novo assembled viral particles to uninfected cells via the virological synapse or filopodia bridge machinery [5-7,23]. To confirm virological synapse formation at the contact site, 293 T cells producing the GALV particles containing YFP labeled gag protein particles (GALV-gag-YFP, as described in Materials and Methods) were used to produce YFP labeled GALV and co-cultured with recipient cells labeled with cell tracker CMAC. CMAC is a fluorescent chloromethyl derivative that can freely diffuse through the membranes of live cells. Once inside the cell, CMAC reacts with intracellular components to produce fluorescent viable cells for at least 24 h. Six to eight hours after co-culture, cells were examined under a confocal microscope. As shown in Figure 7A, accumulation of gag-YFP protein at virological synapses was observed at the contact site of GALV producing 293 T cells (green) and MDTF cells expressing PIT1 (blue), and similar structures were also observed at the contact site of GALV producing cells with MDTF cells expressing mutant receptor (Figure 7B). We did not observe gag-YFP accumulation at the cellular contact sites when GALV-gagYFP producing 293 T cells were co-cultured with MDTF cells expressing PiT2 (data not shown). We observed that a single donor cell, GALV-gag-YFP producing 293 T cell, can contact more than one recipient MDTFPiT1 cell as well as more than one MDTFPiT2K522E cell via a long filopodia-like protrusion observed on donor cells when cocultured with both cell lines; this is marked as a white arrow in Figure 7A. Virological synapses were observed at almost all contact sites when cocultured with MDTFPiT1 recipient cells (Figure 7A), but not at all contact sites with MDTFPiT2K522E cells (Figure 7B). The results indicated that virological synapses or filopodia bridges were formed in cells expressing mutant receptors at a decreased efficiency. Thus two lines of evidence, from co-culture experiments assessing viral transmission and from confocal microscopic analyses, confirmed the reduced ability of productively infected MDTFPiT2K522 cells to efficiently transmit virus in co-culture experiments.

### Syncytium formation is prominent in productively infected cells expressing PiT2K522E

**Figure 8:**
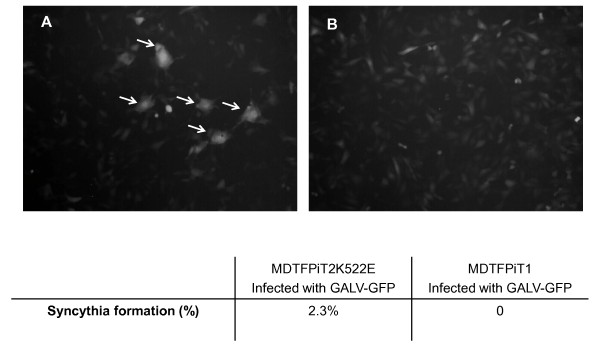
**Syncytia formation observed in MDTFPiT2K522E cells infected with GALV-GFP.** MDTFPiT2K522E (**A**) or MDTFPiT1 cells (**B**) were exposed to GALV-GFP viruses. Four days after viral exposure, Several GFP positive syncytia were observed in MDTFPiT2K522E cells and the number of syncytia gradually increased over time. The experiments were performed three times and the representative images from cells infected with GALV-GFP seven days after viral exposure are shown. Syncytia were defined as those cells containing three or more nuclei. The total number of nuclei in syncythia cells was then counted. Five random fields were counted from each well of triplicate samples, using a 10x objective [11].

We next investigated whether the absence of superinfection interference played a role in the reduced ability of GALV to establish productive infection in MDTFPiT2K522E cells compared with levels demonstrated by GALV employing PiT1 to infect cells. Superinfection has been linked to apoptosis and CPE for several retroviruses [10,12]. We hypothesized that GALV superinfection may contribute to apoptosis that in turn results in diminished viral spread. To test this hypothesis, we utilized PE conjugated Annexin V as well as 7ADD to detect apoptosis in infected cells. Annexin V is a phospholipid binding protein. The appearance of phosphatidylserine (PS) is one of the early structural changes in cells undergoing apoptosis. In the presence of calcium ions, annexin V selectively binds with PS at high affinity. 7ADD is used to examine late cell death. We did not detect significant cell death of GALV-GFP infected cells expressing mutant receptor (data not shown), and thus it does not appear that apoptosis of superinfected cells plays a role in limiting cell spread in GALV infected cells expressing PiT2K522E. However, we did detect a feature that distinguishes cells expressing PiT2K522E from those expressing PiT1 after GALV infection. An increased ability to form syncytia was observed in MDTFPiT2K522E cells productively infected with GALV. Seven days post exposure to GALV, the number of infected MDTFPiT2K522E cells begins to decrease as shown in Figure 1. Concomitant with the initiation of the reduction in the percentage of MDTFPiT2K522E infected cells, is an increase in the number of multinucleated cells demonstrated by comparison of number of syncytia at day 7 in these two infected cell lines (Figure 8). Around 2.3% GALV infected MDTFPiT2K522E were multinucleated (> 3 nuclei/cell), but no syncytia were observed at a similar time point with MDTFPiT1 infected cells nor in the initial days following MDTFPiT2K522E exposure to GALV. Altogether, our results indicate that the decreased affinity of mutant receptor binding to GALV correlates with the failure to productively infect the majority of cells expressing PiT2K522E receptors. Instead of greater than 90% of the cells becoming infected, as is the case for human cells or resistant murine MDTF/CHOK1 cells expressing PiT1, infection of cells expressing PiT2K522E is restricted to approximately 35% of the cell population. This restriction in productive infection also correlates with the diminished ability to establish superinfection resistance, a reduction in the number of virological synapses, and enhanced syncytia formation.

## Discussion

Cell lines derived from Chinese hamster lung fibroblasts such as E36 cells are the only hamster cells susceptible to both GALV and A-MLV. They also express a PiT2 ortholog that functions as a GALV receptor [13]. Cells expressing human PiT2 are not permissive for GALV infection nor does human PiT2 bind GALV RBD or GALV viral particles. Using chimeric E36 hamster PiT2 and human PiT2 proteins, we previously determined that a single residue difference at position 522 of PiT2 accounts for the ability of hamster PiT2 to facilitate GALV as well as A-MLV entry [14]. This difference represents the presence of a glutamate residue in hamster PiT2 in place of the lysine residue present in PiT2. However, we have now determined that even though resistant cells such as murine MDTF cells expressing PiT2K522E, like MDTF cells expressing the human GALV receptor PiT1, are susceptible to GALV vectors, their ability to support infection by replication-competent GALV differ dramatically. In this report, we have attempted to determine what accounts for these differences.

The reduced affinity of the PiT2K522E mutant receptor to GALV compared to wild type PiT1 correlates with a reduced capacity for viral spread (Figures 1 and 2). We have shown that GALV infectious particles produced in cells expressing PiT2K522E are released into the supernatant at levels comparable to PiT1 expressing producer cells (Figure 3). This observation together with the dramatically reduced ability of PiT2K522E to bind GALV envelope suggests that infectious GALV particles are efficiently produced from cells bearing this mutant receptor and that the reduction in cell-to-cell transmission is not mediated by reduced virus production or particle stability. The weak affinity of GALV for target cells bearing PiT2K522E may limit virus spread due to a failure to recruit sufficient levels of receptor and induce conformation changes necessary to mediate viral uptake via viral synapse formation [24]. The most efficient means of retrovirus spread following exposure to cell-free virus involves the transfer of viral particles from infected cells to uninfected target cells via viral synapses formed at the interface of these two types of cells [6,7,23-25]. GALV producer cells co-cultured with MDTFPiT1 efficiently triggered the formation of virological synapse mediating the apparent transfer of virus from one donor to, on average, three recipient cells in co-culture experiments. Similar experiments carried out with PiT2K522E expressing recipient cells showed a reduction in the ratio of donor to recipient cells (e.g., 1:1) and a reduction in synapse formation (Figure 7). Efficient cell-cell transmission is dependent on the formation of virological synapses or filopodia bridges that, in turn, depends on virus being efficiently maintained on the cell surface by receptors prior to extracellular release or retained in cells that fail to release mature viral particles [24]. Cells expressing PiT2K522E form virological synapses or filopodia bridges less efficiently than cells expressing PiT1and this may account for the reduced levels of productively infected cells (Figure 1). We have shown previously, that cell-free GALV pseudotyped retroviral vectors transduce cells expressing PiT2K522E five times less efficiently than cells expressing PiT1 [16]. The results presented here show that cell to cell transmission is also diminished in PiT2K522K infected cells indicating that reduced receptor binding affinity affects both GALV cell-free and cell to cell transmission.

Altered envelope receptor binding kinetics attributed to specific residue changes in the envelope has been shown to affect the cell fusion process and the spectrum of disease associated with ecotropic murine and feline retroviruses. It has been reported that adaptive changes in the envelope protein of gammaretroviruses that confer changes in receptor binding properties (compared to their prototypic equivalents) correlate with altered disease properties. A recent report characterized a feline leukemia virus subgroup A (FeLV A) isolate, FeLV-945, that causes an uncharacteristic disease and demonstrated that it has a significantly greater receptor binding capacity than the prototypic FeLV-A, FeLV61E [26]. It was proposed that this enhanced receptor affinity might serve to increase the kinetics of virus spread and render target cells with reduced receptor numbers susceptible to infection *in vivo* thereby altering the disease status of infected cats. At the opposite end of the spectrum, a finding similar to the results presented here, has been reported by S.L. Murphy *et al*. [10]. This group found a linkage of specific residue changes in the ecotropic MLV ENV (TR1.3) that lead to decreased receptor affinity, the loss of superinfection resistance, syncytium formation [27] and attendant pathology.

In this report, we demonstrate that a single virus, GALV, can exhibit distinct receptor affinity, superinfection interference properties, syncytium formation and viral spreading capacities depending on the receptor that is employed to enter cells. Thus the receptor ortholog or the envelope protein can mediate dramatic differences in receptor dependent properties with as little as a single residue difference as shown in this paper and previous studies [26]. The cell lines expressing low affinity and high affinity GALV receptors (MDTFPiT2K522E and MDTFPiT1, respectively) employed in the analysis of viral infection and spread are useful tools in dissecting specific stages critical to cell-cell transmission. These cells should prove valuable for *in vitro* testing of reagents designed to prevent cell mediated viral spread.

## Methods

### Cell culture

Cell lines used in this study include *Mus dunni* tail fibroblasts MDTF [17], Chinese hamster ovary (CHOK1) cells, CCL61, (ATCC), and 293 T (formerly referred to as TSA cells) [28]. All cells, with the exception of CHOK1, were maintained in Dulbecco’s modified Eagle’s medium with Glutamax (DMEM) (Invitrogen), supplemented with 10% fetal bovine serum, 100 units of penicillin/ml, and 100 ug/ml of streptomycin. CHOK1 cells were maintained in alpha minimal essential medium (MEM) supplemented with 10% fetal bovine serum, 100 units/ml, of penicillin and 100 ug/ml of streptomycin (Invitrogen). The GALV viral receptor PiT1, A-MLV viral receptor PiT2 and mutant receptor PiT2K522E all tagged with a hemagglutinin (HA) eptitope were generated as previously described [16]. Vesicular stomatitis virus (VSV) G protein-enveloped retroviral vector pLNSX [29] expressing the appropriate receptors was used to transduce respective cell lines and G418 allowing selection of cells stably expressing these receptors.

### Plasmids

The GALV-GZAP (generously provided by Christopher Logg, University of California, Los Angeles, CA,) is replication-competent ecotropic Moloney MLV backbone in which the GALV env gene replaces the MLV env gene. In addition an IRES driven GFP (IRES-GFP) is positioned between the 3′terminus of the env gene and untranslated region of 3′LTR [29]. The GALV-GZAP variant, MSA2-GFP [18] contains an insertion of TCC at the MLV splice acceptor and was shown to replicate more efficiently than GALV-GZAP and was thus used as the GFP expressing GALV enveloped virus in this study. We further modified the MSA2-GFP plasmid removing the IRES-GFP fragment and this construct was used to produce GALV enveloped virus that does not express GFP. The GALV RBD fragment was inserted into the pcDNA3.1-V5-His B plasmid (Invitrogen). Fragments encoding envelope residues 1 to 219 of GALV RBD were amplified by PCR from the GALV envelope subclone [16] using primers containing EcoRI and XbaI at their 5′ and 3′ termini, respectively. The EcoRI and XbaI-digested PCR product was ligated into the pcDNA3.1-V5-His B plasmid. To construct a vector genome expressing fluorescent cherry red protein, RFP, the GFP gene in the retroviral vector, pRT43.2 GFP [30] was removed by digesting with PmlI (blunted) and NotI and this fragment was removed and replaced with RFP gene that was obtained from CMV-brainbow 1.1 M plasmid (Addgene, Inc.) cleaved by BglII (blunted) and NotI. All constructs generated by PCR were verified by nucleotide sequence analyses.

### Production of replication-competent retroviruses, retroviral vectors and GALV RBD

The GALV enveloped replication-competent retroviruses (GALV-GFP and GALV), GALV enveloped retroviral vectors as well as GALV RBD were produced by calcium phosphate transfection of 293 T cells (Promega Corporation) as previously described [31]. Supernatants were harvested 48 to 72 h post-transfection, then passed through a 0.45 mM Millipore (Millipore Corporation) and stored at −80 °C. GALV RBD purification was performed following manufacturer’s instruction (Invitrogen) with modifications. Supernatants that contain GALV RBD tagged with V5 and 6xHis epitope were concentrated greater than 10-fold using Amicon Ultra-15, centrifugal filters (Millipore Corporation), then mixed with 40 ml binding buffer (1x Hanks Balanced Salt Solution (HBSS) (Mediatech, Inc.) containing 10 mM imidazole (ACROS). Five ml samples were loaded onto the high-performance nickel-NTA (Ni-NTA) agarose column (Invitrogen), unbound proteins were removed with washing buffer (1x HBSS containing 20 mM imidazole) and bound GALV RBD was eluted with buffer (1x HBSS containing 250 mM imidazole) and collected. The concentrated GALV RBD was passed through Econo-Pac 10DG desalting column to remove imidazole (BioRad Life Science). The purified GALV RBD was quantified using the BCA protein assay kit (Pierce) and stored in aliquots at −80 °C.

### Co-culture of GALV infected cells with target cells

MDTFPiT1 or MDTFPiT2K522E cells were exposed to replication-competent GALV-GFP viruses. One week after initial exposure, infected cells were mixed with an equivalent number of uninfected MDTFPiT1-HA or MDTFPiT2K522E-HA cells. Thirty hours after co-culture, HA-tagged receptors were detected on the cell surface by incubation of MDTF cells expressing receptors with monoclonal HA antibody HA.11 (Covance Inc.), followed by incubation with a RPE conjugated secondary antibody (Santa Cruz biotechnology) and then analyzed by flow cytometry.

### Production of GALV-gag-YFP viruses and visualization of virological synapses

Approximately 2×10^5^ 293 T cells were exposed to replication-competent GALV viruses for one week, grown overnight on a 35 mm tissue culture dish with a 0.17 mm thick glass bottom, and then transfected with 0.2ug MLV-gag-YFP plasmid. MLV-gag-YFP consists of MLV gag protein fused in frame to YFP at the nucleotides encoding the residues PQ at present at the C-terminus of its gag nucleocapsid domain (Addgene Inc.). Sixteen hours later, these cells were incubated with 2×10^5^ uninfected MDTFPiT1-HA, MDTFPiT2K522E-HA or MDTFPiT2-HA cells labeled with CMAC cell tracker blue (Invitrogen) according to manufacturer’s instructions. The *de novo* synthesized gag-YFP proteins in the infected 293 T cells were recruited and used in GALV assembly to produce GALV virions that contain YFP labeled gag at cell-cell contact site. Six hours after co-culture at 37 °C, cells were fixed with 2% paraformadehyde in PBS solution and visualized on an LSM510 inverted Meta confocal microscope (Carl Zeiss, Thornwood, NY) with a 63× 1.4 NA oil immersion objective. CMAC was excited with a 405 nm laser, YFP with the 514 nm line of a krypton/argon laser. YFP was imaged with a 530–600 nm bandpass filter, CMAC with a 420–500 nm bandpass filter.

### Flow cytometry

Epics XL (Beckman Coulter, Fullerton, CA) and FACScan (Becton Dickinson, Franklin Lakes, NJ) flow cytometers were used to assess expression of GFP, RFP, HA and V5 tagged proteins in infected and transfected cells using a 525 nm or a 530 nm band pass emission filter. 20,000 cells from each sample were analyzed after trypsinization and suspension in HBSS. Propidium iodide (Sigma) was added to cell suspension at a concentration of 1ug/ml to exclude dead cells in FACS analysis.

## Competing interests

The authors declare that they have no competing interests.

## Authors’ contributions

ML conducted experiments, ML and MVE evaluated data and designed experiments and wrote the manuscript. Both authors read and approved the final manuscript.

## Supplementary Material

Additional file 1 **Figure S1.** The expression PiT2K522E on productively GALV-GFP infected MDTF cells expressing PiT2K522E tagged with HA epitope. A. Uninfected MDTFPiT2K522E-HA cells. B. MDTFPiT2K522E cells transduced with GALV-GFP viruses three weeks after initial viral exposure. The cells were unstained (left) or stained (right) with anti-HA monoclonal antibodies and isotope specific secondary antibody conjugated with R-phyoerythrinan and analyzed by flow cytometry. The x-axis represents MFI of GFP expression and the y-axis represents MFI of HA expression on the cell surface. The experiment was performed three independent times, and images are from one representative experiment. (PPT 176 kb) Click here for file
