# Supplementary material for: A mutant retroviral receptor restricts virus superinfection interference and productive infection
Source: Retrovirology. 2012 Jun 12;9:51. doi: 10.1186/1742-4690-9-51 (PMC3418563; doi:10.1186/1742-4690-9-51)
Supplement: Additional file 1 — Figure S1. The expression PiT2K522E on productively GALV-GFP infected MDTF cells expressing PiT2K522E tagged with HA epitope. A. Uninfected MDTFPiT2K522E-HA cells. B. MDTFPiT2K522E cells transduced with GALV-GFP viruses three weeks after initial viral exposure. The cells were unstained (left) or stained (right) with anti-HA monoclonal antibodies and isotope specific secondary antibody conjugated with R-phyoerythrinan and analyzed by flow cytometry. The x-axis represents MFI of GFP expression and the y-axis represents MFI of HA expression on the cell surface. The experiment was performed three independent times, and images are from one representative experiment. (PPT 176 kb) [file 1742-4690-9-51-S1.ppt]

## Slide 1
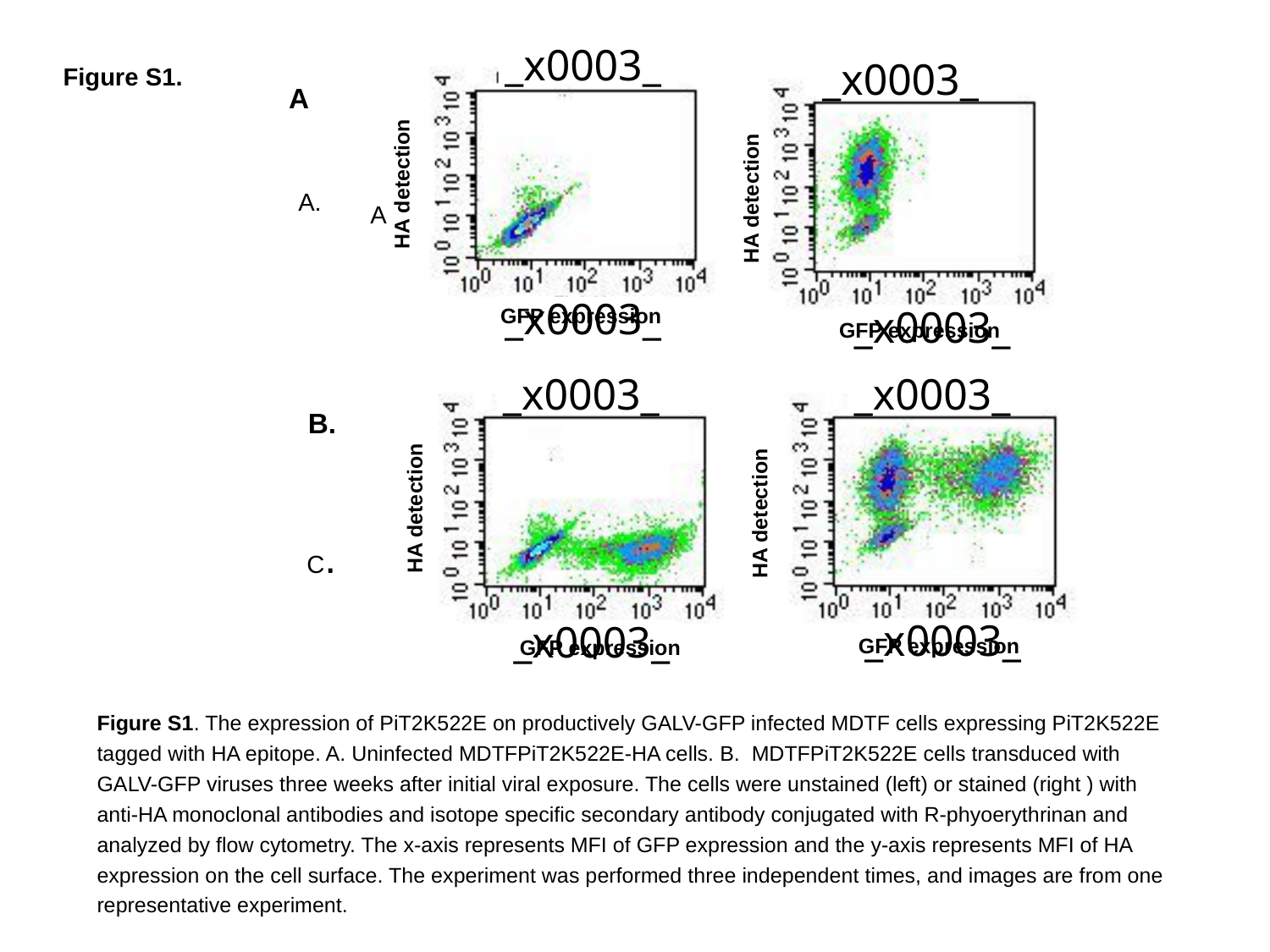

_x0003_
_x0003_
HA detection
GFP expression
_x0003_
_x0003_
HA detection
GFP expression
A
Figure S1.
B.
A.
A
_x0003_
_x0003_
HA detection
GFP expression
_x0003_
_x0003_
HA detection
GFP expression
B.
C.
D.
Figure S1. The expression of PiT2K522E on productively GALV-GFP infected MDTF cells expressing PiT2K522E tagged with HA epitope. A. Uninfected MDTFPiT2K522E-HA cells. B. MDTFPiT2K522E cells transduced with GALV-GFP viruses three weeks after initial viral exposure. The cells were unstained (left) or stained (right ) with anti-HA monoclonal antibodies and isotope specific secondary antibody conjugated with R-phyoerythrinan and analyzed by flow cytometry. The x-axis represents MFI of GFP expression and the y-axis represents MFI of HA expression on the cell surface. The experiment was performed three independent times, and images are from one representative experiment.
